# The utility of mini-Clinical Evaluation Exercise (mini-CEX) in undergraduate and postgraduate medical education: protocol for a systematic review

**DOI:** 10.1186/s13643-017-0539-y

**Published:** 2017-07-18

**Authors:** Sara Mortaz Hejri, Mohammad Jalili, Mandana Shirazi, Rasoul Masoomi, Saharnaz Nedjat, John Norcini

**Affiliations:** 1 Department of Medical Education, Health Professions Education Research Center, Tehran University of Medical Sciences, Tehran, Iran; 2 Department of Medical Education, Department of Emergency Medicine, Tehran University of Medical Sciences, 57, Hojjatdoust St, Keshavarz Blvd, Tehran, Iran; 3 Education Development Center, Department of Medical Education, Tehran University of Medical Sciences, Tehran, Iran; 4 Department of Clinical Science and Education at SOS Hospital, Karolina Institute, Stockholm, Sweden; 5 Department of Medical Education, Tehran University of Medical Sciences, Tehran, Iran; 6 Department of Epidemiology and Biostatistics, School of Public Health, Tehran University of Medical Sciences, Tehran, Iran; 7 Foundation for Advancement of International Medical Education and Research (FAIMER), Philadelphia, PA USA

**Keywords:** Systematic review, Workplace-based assessment, mini-CEX

## Abstract

**Background:**

One of the most frequently used assessment tools that measure the trainees’ performance in workplace is the mini-Clinical Evaluation Exercise (mini-CEX), in which an expert observes and rates the actual performance of trainees. Several primary studies have evaluated the effectiveness of mini-CEX by assessing its educational and psychometric properties. The objective of this BEME review is to explore, analyze, and synthesize the evidence considering the utility of the mini-CEX for assessing undergraduate and postgraduate medical trainees.

**Methods:**

Studies reporting on mini-CEX performed in undergraduate and postgraduate medical education and providing some empirical data for mini-CEX in relation to one or more of the validity, reliability, educational impact, acceptability, and cost of mini-CEX will be included in the review. No restrictions on study design or publication date or language will be handled. To ensure comprehensiveness of our search, we will use different approaches and methods. In addition to electronic search in bibliographic databases, we will conduct forward and backward searching. We will also contact leading authors in the field of mini-CEX and will search for the gray literature. Data extractions will be done independently by two coders based on a form. If there is any discordance, a third author will resolve it.

The quality assessment will be also done independently by two team members, based on critical appraisal checklists. In attempting to answer our original research questions, we will use meta-analysis or meta-synthesis.

**Discussion:**

The findings of this study can be transferred to the medical education stakeholders such as administrators of medical schools, residency program directors, and faculty members.

We also hope that publication of this review will encourage stakeholders who have already adopted the mini-CEX to evaluate and report its different characteristics. Lastly, we expect that we can identify gap of knowledge in this field and suggest areas for future research.

## Background

### Role of assessment

Assessment plays a central role in medical curriculum. It completes learning process by measuring students’ progress and achievement regarding the curriculum outcomes. Several tools have been developed for serving this purpose. Some of these methods focus on cognitive domain of learning and require students to present their knowledge in basic or clinical sciences in written or oral exams, while new assessment methods emphasizes on assessing students’ clinical skills, either in a simulated setting dealing with standardized patients or in a workplace setting encountering the actual patients.

### mini-CEX

One of the most frequently used assessment tools that measure the trainees’ performance in workplace is the mini-Clinical Evaluation Exercise (mini-CEX). In its original form, the mini-CEX is a 9-point rating scale organized in three levels of unsatisfactory (1–3), satisfactory (4–6), and high satisfactory (7–9). An expert, usually a faculty member, observes the actual performance of trainees, rates their history taking and physical examination skills, and provides feedback to them [1]. Often, it is required that different experts rate several clinical encounters of a trainee throughout the course, rather than one single occasion to be observed by one individual rater.

Following development of the mini-CEX by the American Board of Internal Medicine (ABIM) in the 1990s, it has been widely used in undergraduate and postgraduate medical education programs around the world [2], both for formative and summative purposes [3].

### Utility

A common framework used to explore “all” aspects of assessment tools is the utility formula proposed by van der Vleuten in 1996. Several individual studies and systematic reviews have applied this framework to discuss the effectiveness of different assessment tools [4, 5]. The framework is composed of validity, reliability, educational impact, acceptability, and the cost of the assessment tool [6].

### Scoping review of the research on mini-CEX

We decided to conduct a scoping search in MEDLINE (via Ovid) to help us make decisions on the final search strategy. The results have been presented in Table 1.

**Table 1:** Scoping search for MEDLINE (via Ovid)

Terms		Number of retrieved titles
# 1	mini-CEX.mp.	120
# 2	mCEX.mp.	7
# 3	miniCEX.mp.	4
# 4	mini-clinical evaluation exercise*.mp.	92
# 5	miniclinical evaluation exercise*.mp.	3
# 6	clinical evaluation exercise*.mp.	124
# 7	mini clinical exam*.mp.	14
# 8	direct observation of clinical skill*.mp.	8
# 9	workplace based assessment*.mp.	228
# 10	work based assessment*.mp.	52
# 11	work place based assessment*.mp.	6
# 12	1 or 2 or 3 or 4 or 5 or 6 or 7 or 8 or 9 or 10 or 11	426

Database: Ovid MEDLINE(R) In-Process & Other Non-Indexed Citations, Ovid MEDLINE(R) Daily, and Ovid MEDLINE(R) 1946 to present; search date: 30 September 2016

### Lessons learned from the scoping review

We found several primary studies evaluating the effectiveness of mini-CEX by assessing its educational and psychometric properties. Conducting the scoping review helped us learn a couple of lessons which were useful for developing this protocol and performing the review in future. We realized that different studies have used various keywords for the instrument, including mini-CEX, mCEX, DOCS, and mini-Clinical Exam. We also noticed that the studies have targeted different populations (undergraduate, post-graduates, practicing physician, and so on) and have been conducted in different contexts (pediatrics, emergency medicine, etc.). The studies also varied in several other aspects: either original or modified version of the mini-CEX form has been used in different studies; the tool has been used for different purposes (formative vs. summative assessment); different numbers of encounters have been considered adequate; raters have been different (faculty members, senior residents, etc.); the length of rotation in which mini-CEX was used varied; various numbers of forms have been filled for each learner; and finally, different outcomes have been evaluated. We used these lessons later in developing our search strategy and also incorporated these findings in our data extraction form.

Also, we found a number of systematic reviews that have tackled the issue of workplace-based assessment (WPBA) tools. While some of them included mini-CEX besides other WPBA tools, three reviews have focused solely on the mini-CEX. One is a literature review by Hawkins et al. which analyzed the mini-CEX scores within a validity argument framework in terms of scoring, generalization, extrapolation, and interpretation/decision. The search was conducted in MEDLINE only, and the inclusion criteria were the investigations that had used all or some of the items on the mini-CEX scale without significant changes in scale structure or item descriptors. So, instruments that were significantly modified from the original format had been excluded. The authors stated that except for the area of scoring, other components of the argument generally appeared to be supportive [7]. Another work is a meta-analysis by Ansari et al. aimed at determining the construct and criterion validity of the mini-CEX. The reviewers evaluated the use of the mini-CEX to assess medical students’ or residents’ clinical skills in comparison with those participants’ use of other clinical measures at various training levels. They included studies that had used the original seven-item version of the mini-CEX and had been published in a peer-reviewed journal. Papers that had focused on generalizability analysis or investigation of the internal structure of the mini-CEX were excluded from the meta-analysis [8]. Another systematic review conducted by Sandilands and Zumbo discusses validity theory and validation practices. They restricted their search to the literature published in English language. Articles whose main purposes were not only to investigate other assessment tools but also to mention mini-CEX were excluded from the review. The reviewers found 13 studies that had investigated the validity of the mini-CEX. However, the authors emphasized that, by conducting this review, they wanted to provide an example to show the (mis)alignment of perspective in current research with the contemporary validity theory, rather than evaluating the quality of the mini-CEX [9].

As noted, each of the abovementioned systematic reviews focused on one or more characteristics of the mini-CEX. In other words, none of the studies adopted a comprehensive framework to analyze its utility. Since van der Vleuten utility framework provides useful and comprehensive criteria beyond traditional psychometrics of validity and reliability, in this systematic review, we are going to evaluate the mini-CEX as a method of assessment in undergraduate and postgraduate medical education, using van der Vleuten formula for utility in order to investigate all abovementioned characteristics.

## Methods

### Review questions, objectives, and keywords

The main objective of this Best Evidence Medical Education (BEME) review is to explore, analyze, and synthesize the evidence considering the utility of the mini-CEX for assessing undergraduate and postgraduate medical trainees.

The specific objectives are to:

- Explore, analyze, and synthesize the evidence considering the “validity” of the mini-CEX for assessing undergraduate and postgraduate medical trainees
- Explore, analyze, and synthesize the evidence considering the “reliability” of the mini-CEX for assessing undergraduate and postgraduate medical trainees
- Explore, analyze, and synthesize the evidence considering the “educational impact” of the mini-CEX for assessing undergraduate and postgraduate medical trainees
- Explore, analyze, and synthesize the evidence considering the “cost” of the mini-CEX for assessing undergraduate and postgraduate medical trainees
- Explore, analyze, and synthesize the evidence considering the “acceptability” of the mini-CEX for assessing undergraduate and postgraduate medical trainees

Hence, the following research questions have been developed:

- What is the validity evidence for mini-CEX in the assessment of undergraduate and postgraduate medical trainees?
- What is the reliability of mini-CEX in the assessment of undergraduate and postgraduate medical trainees?
- What is the educational impact of mini-CEX on the undergraduate and postgraduate medical trainees?
- What is the cost of using mini-CEX in the assessment of undergraduate and postgraduate medical trainees?
- How acceptable is mini-CEX to medical students and faculty members in undergraduate and postgraduate settings?

Considering the results of our scoping search, we have identified the following keywords for this systematic review:

Population:

- Undergraduate medical trainee, Undergraduate medical education, basic medical education, Medical student
- Postgraduate medical trainees, graduate medical education, residency training, resident

Activity:

- mini-Clinical Evaluation Exercise, Mini-CEX, mCEX, Direct Observation of Clinical Skills, DOCS, Clinical Evaluation Exercise, CEX

Outcome:

- Utility
- Validity, credibility
- Reliability, generalizability, reproducibility, consistency, accuracy
- Educational impact, educational effect, learning impact, educational outcome, consecutive validity
- Cost, cost-effectiveness, feasibility
- Acceptability

For the purpose of this study, the abovementioned keywords would be defined as follows:

- Undergraduate medical trainees are students undertaking undergraduate or basic medical education at a medical school in order to reach a primary qualification in medicine.
- Postgraduate medical trainees are learners of educational programs for medical graduates entering a specialty. They include formal specialty training as well as academic work in the clinical sciences.
- mini-CEX or mini-Clinical Evaluation Exercise is an assessment tools used by supervisors in workplace settings to assess clinical performance and provide feedback on a direct observation basis.
- Utility is composed of five components including validity, reliability, educational impact, acceptability, and cost.

### Study selection criteria

The inclusion/exclusion criteria have been summarized in Table 2.

**Table 2:** Inclusion and exclusion criteria

	Inclusion criteria	Exclusion criteria
Population	Studies on undergraduate or postgraduate medical trainees	Studies on non-medical students, studies on CME/CPD
Activity	Studies on the mini-CEX	Studies on OSCE, DOPS, MSF, or CbD
Outcome	Studies providing data on validity, reliability, educational impact, acceptability, or cost	
Study language	All languages	No limitation
Study type	All designs	Papers describing non-primary empirical research (letters and editorial papers)

Studies reporting on mini-CEX performed in undergraduate and postgraduate medical education and providing some empirical data for mini-CEX in relation to one or more of the validity, reliability, educational impact, acceptability, and cost of mini-CEX will be included in the review.

In addition to the original forms of the mini-CEX, modified versions will also be included in the review, but a subgroup analysis would be done, if needed.

The studies reported multiple data regarding a variety of WPBA tools, only included if mini-CEX data would have been reported separately.

No restrictions on study design or publication date or language will be handled.

The following studies will be excluded:

- Studies on non-medical trainees
- Studies on Continuing Medical Education (CME)/Continuing Professional Development (CPD)
- Studies describing non-primary empirical research
- Studies on other WPBA tools such as:

- ○ Objective Structured Clinical Examination (OSCE)
- ○ Direct Observation of Procedural Skills (DOPS)
- ○ Multi-Source Feedback (MSF)
- ○ Case-based Discussion (CbD)

Two reviewers will select the studies according to the inclusion/exclusion criteria, independently. In case of any discordance, the reviewers will discuss the issue, and if it is needed, a third reviewer will be consulted.

At the end, the consistency between coders will be checked by calculating kappa coefficient for each item.

### Search sources and strategies

To ensure comprehensiveness of our search, we will use different approaches and methods. Regarding electronic search, the following bibliographic databases will be explored: MEDLINE/PubMed, EMBASE, CINAHL, ERIC, PsycINFO, SCOPUS, and Web of Sciences. In order to find the gray literature, we will search ProQuest Dissertations & Theses and OpenGrey. In addition, we will conduct forward and backward searching by checking the reference lists and citations of the included articles and review articles for additional relevant studies. We will also contact leading authors in the field of mini-CEX.

According to the fact that less than 500 studies were found in our scoping search, we assumed that including population (i.e., undergraduate and postgraduate medical trainees), together with study outcomes (reliability, validity, etc.), would diminish the sensitivity of search strategy and limit the number of retrieved articles even further.

### Procedure for extracting data

In order to extract and present data from the primary studies, we have designed a data extraction form as can be seen in Table 3:

**Table 3:** The primary version of the data extraction form

Paper code	First author name	Publication date	Journal	Study design	Country	Postgraduate or undergraduate trainees	Sample size	mini-CEX characteristics	Frequency of use	Summative or formative purposes	Validity	Reliability	Educational impact	Acceptability	Cost	Study quality
001																
002																
003																
004

- The extraction form might be revised after checking the selected articles. Before starting the extraction, we will check the reliability of this form. So, two authors independently review ten articles using this form, and the kappa coefficient will be calculated for each item. The items with kappa less than 0.7 will be modified or rechecked. Then, the final form will be approved.
- All the extractions will be done independently by two coders based on the final form. If there is any discordance, since it is possible that the source of the disagreement is an error by one side, in the first place, the coders will be asked to discuss the issue. However, if the disagreement cannot be resolved at this stage, a third reviewer will be consulted. He/she will independently extract data and then discuss with two coders to reach consensus. If consensus cannot be reached, the study authors’ will be contacted for further information, and the final decision will be made.
- At the end, the consistency between coders will be checked by calculating kappa coefficient for each item.

### Study quality assessment

To evaluate the methodological quality of quantitative studies, we will use the BEME quality framework which consists of 11 indicators [10]. Each indicator will be rated as “met,” “unmet,” or “unclear.” In order to be deemed of high quality, studies should meet a minimum of seven indicators (Table 4).

**Table 4:** BEME quality indicators [10]

No.	Category	Question
1.	Research question	Is the research question or hypothesis clearly stated?
2.	Study subjects	Is the subject group appropriate for the study being carried out?
3.	Data collection methods	Are the methods used appropriate for the research question and context?
4.	Completeness of data	Attrition rates/acceptable questionnaire response rates
5.	Risk of bias assessment	Is a statement of author positionality and a risk of bias assessment included?
6.	Analysis of results	Are the statistical and other methods of results analysis used appropriate?
7.	Conclusions	Is it clear that the data justify the conclusions drawn?
8.	Reproducibility	Could the study be repeated by other researchers?
9.	Prospective	Is the study prospective?
10.	Ethical issues	Are all ethical issues articulated and managed appropriately?
11.	Triangulation	Were results supported by data from more than one source?

To evaluate the methodological quality of qualitative studies, the Critical Appraisal Skills Program (CASP) checklist for the reporting of all qualitative studies will be applied (Table 5). The coder will be asked to record a “Yes”, “No”, or “Can’t tell” response.

**Table 5:** The Critical Appraisal Skills Program (CASP) checklist for quality assessment of qualitative studies

No.	Question
1.	Was there a clear statement of the aims of the research?
2.	Is a qualitative methodology appropriate?
3.	Was the research design appropriate to address the aims of the research?
4.	Was the recruitment strategy appropriate to the aims of the research?
5.	Was the data collected in a way that addressed the research issue?
6.	Has the relationship between researcher participants been adequately considered?
7.	Have ethical issues been taken into consideration
8.	Was the data analysis sufficiently rigorous?
9.	Is there a clear statement of findings?
10.	How valuable is the research

These checklists will guide the team members for deciding about the quality of the articles and might be completed or tailored according to the included studies’ type.

The quality assessment will be done independently by two team members. Disagreements, similar to the aforementioned procedure, will be resolved via discussion between two coders, and, if required, a third reviewer’s opinion will be sought.

We will not remove any study due to weak methodology, though we will consider the quality for quantitative subgroup analysis (if the meta-analysis is possible) or narrative synthesis.

### Synthesis of extracted evidence

First, we will provide a description on the characteristics, setting, and context of the included studies. This descriptive synthesis will be used as the basis of synthesis evidence in order to address the review questions and objectives.

In attempting to answer our original research questions, we will synthesize the findings to discuss the utility of mini-CEX in undergraduate and postgraduate medical training. We will present and discuss our findings according to five outcomes (validity, reliability, acceptability, educational impact, and cost).

It is expected to find quantitative data mostly for questions concerning reliability and cost of the mini-CEX. Similarly, it is supposed to have both qualitative and quantitative data for questions regarding validity, educational impact, and acceptability.

Considering quantitative data, we predict that there would be significant heterogeneity among the studies that preclude meta-analysis. It means that due to variation in studies’ setting, design, and methodology meta-analysis would not be appropriate. However, we will test the statistical heterogeneity by *I* ^2^ and *χ* ^2^ and check it visually through forest plot. If the findings can be quantitatively synthesized, the random-effects model and subgroup analysis (regarding the study quality and study population) will be applied. Otherwise, if there is heterogeneity, we will report the study findings narratively and will undertake a rich and exploratory descriptive synthesis of evidence to explain differences in findings.

Publication bias will be checked through funnel plot and Begg’s or Egger’s tests for relevant outcomes like educational impact.

Regarding qualitative data, we will use meta-synthesis. Meta-synthesis is an evolving methodology developed to enable systematic synthesis of qualitative data which allows researchers to make explicit the layers of interpretation. We will explore iterative themes within the data and deductively address some of the research questions. As mentioned earlier, a number of our research questions and objectives will be addressed by this approach.

This protocol has not been registered with PROSPERO.

## Discussion

The findings of this study can be transferred to the medical education stakeholders such as administrators of medical schools, residency program directors, and faculty members through brief reports (1:3:25 format) or discussion meetings.

Direct observation of medical trainees who deal with actual patients in real workplace is important for performance-based assessment. We are going to evaluate the utility of mini-CEX in the light of the existing literature through a systematic approach. If the mini-CEX proves to be useful in evaluating the performance of medical trainees, it can be recommended as part of the formative or even summative assessment in educational programs.

We also hope that publication of this review will encourage stakeholders who have already adopted the mini-CEX to evaluate and report its different characteristics. Lastly, we expect that by conducting a thorough analysis of the psychometric properties of this instrument, we can identify gap of knowledge in this field and suggest areas for future research.

## Abbreviations

ABIM: American Board of Internal Medicine
CASP: Critical Appraisal Skills Program
CbD: Case-based Discussion
CME: Continuing Medical Education
CPD: Continuing Professional Development
DOPS: Direct Observation of Procedural Skills
mini-CEX: mini-Clinical Evaluation Exercise
MSF: Multi-Source Feedback
WPBA: Workplace-based assessment
